# Severe *Plasmodium knowlesi* Malaria in a Tertiary Care Hospital, Sabah, Malaysia

**DOI:** 10.3201/eid.1707.101017

**Published:** 2011-07

**Authors:** Timothy William, Jayaram Menon, Giri Rajahram, Leslie Chan, Gordon Ma, Samantha Donaldson, Serena Khoo, Charlie Fredrick, Jenarun Jelip, Nicholas M. Anstey, Tsin Wen Yeo

**Affiliations:** Author affiliations: Queen Elizabeth Hospital, Kota Kinabalu, Sabah, Malaysian Borneo (T. William, J. Menon, G. Rajahram, L. Chan, G. Ma, S. Donaldson, S. Khoo, C. Fredrick);; Department of Health, Kota Kinabalu, Malaysia (J. Jelip);; Menzies School of Health Research and Charles Darwin University, Darwin, Northern Territory, Australia (N.M. Anstey, T.W. Yeo);; Royal Darwin Hospital, Darwin (N.M. Anstey, T.W. Yeo)

**Keywords:** Severe malaria, Plasmodium knowlesi, artemisinin derivatives, artesunate, Sabab, Malaysian Borneo, parasites, research

## Abstract

The simian parasite *Plasmodium knowlesi* causes severe human malaria; the optimal treatment remains unknown. We describe the clinical features, disease spectrum, and response to antimalarial chemotherapy, including artemether-lumefantrine and artesunate, in patients with *P. knowlesi* malaria diagnosed by PCR during December 2007–November 2009 at a tertiary care hospital in Sabah, Malaysia. Fifty-six patients had PCR-confirmed *P. knowlesi* monoinfection and clinical records available for review. Twenty-two (39%) had severe malaria; of these, 6 (27%) died. Thirteen (59%) had respiratory distress; 12 (55%), acute renal failure; and 12, shock. None experienced coma. Patients with uncomplicated disease received chloroquine, quinine, or artemether-lumefantrine, and those with severe disease received intravenous quinine or artesunate. Parasite clearance times were 1–2 days shorter with either artemether-lumefantrine or artesunate treatment. *P. knowlesi* is a major cause of severe and fatal malaria in Sabah. Artemisinin derivatives rapidly clear parasitemia and are efficacious in treating uncomplicated and severe knowlesi malaria.

The simian parasite *Plasmodium knowlesi* has recently been found to be a major cause of human malaria in Malaysian Borneo (*1**,**2*), with the disease also reported from southern and eastern Asia (*3*). To our knowledge, the only large epidemiologic and clinical studies have been from Sarawak State, Malaysian Borneo, with case series or reports from persons or returning travelers from Myanmar (*4*), Thailand (*5**,**6*), Vietnam (*7*), Philippines (*8**,**9*), Singapore (*10*), Sarawak (*11*), western Malaysia (*12*), and Indonesia (*13*).

The potential for *P. knowlesi* to cause severe disease has been suggested by experimental simian and human infections (*14**–**16*). The first description of naturally acquired severe human *P. knowlesi* infection was a retrospective study from Sarawak that detailed 4 fatal cases with multiorgan failure (*17*). Subsequently, a prospective study from the Kapit District Hospital in Sarawak enrolled 107 persons with *P. knowlesi* monoinfection and demonstrated that 10 patients had severe disease as defined by World Health Organization (WHO) criteria, resulting in 2 deaths (*2*).

The disease spectrum and clinical features of large numbers of patients infected with *P. knowlesi* have not been described outside Sarawak. To reliably differentiate *P. malariae* from *P. knowlesi* infections by using only microscopy is difficult (*18*); such differentiation requires molecular methods (*1*). In a random survey from several districts in Sabah, the state that borders Sarawak, 44 of 49 cases of microscopy-diagnosed *P. malariae* infection were confirmed by PCR to be *P. knowlesi*, indicating that knowlesi malaria was not confined to isolated areas (*17*). In recent years at Queen Elizabeth Hospital (QEH), a tertiary care referral hospital in Kota Kinabalu, Sabah State, patients with severe malaria by WHO criteria had received a diagnosis by microscopy as *P. malariae* infection, but *P. knowlesi* was suspected as the etiologic agent. We conducted a retrospective review of the clinical spectrum of all case-patients with *P. malariae* malaria who were admitted to QEH during December 2007–November 2009 and confirmed the diagnosis of *P. malariae* or *P. knowlesi* infection by molecular methods.

The optimal management of knowlesi malaria is not known. *P. knowlesi* infection has been successfully treated with chloroquine (*2*) and quinine (*2*); however, the therapeutic efficacy of other antimalarial agents is not known. Artemisinin-derivative combination therapy is now the WHO treatment of choice for uncomplicated falciparum malaria (*19*) and is increasingly recommended for nonfalciparum malaria (*20*); its efficacy in knowlesi malaria is unknown. Similarly, intravenous artesunate is now the treatment of choice for severe falciparum malaria in adults (*19**,**21*), but the therapeutic response to this regimen in severe knowlesi malaria is unknown. As part of our study, we documented the therapeutic responses in uncomplicated and severe knowlesi malaria treated with artemisinin derivatives.

## Methods

### Study Site

QEH serves as a tertiary care hospital for the Malaysian state of Sabah, which has an estimated population of 3 million. It has a modern well-equipped intensive care unit with facilities for invasive ventilation, hemodynamic support, and renal replacement therapy.

### Retrospective Case Review

All patients with microscopy-diagnosed malaria during December 2007–November 2009 were recorded from a prospective laboratory register, and those with *P. malariae* monoinfection or mixed infections were identified. Additional patients, for whom conditions had been diagnosed by microscopy as caused by other *Plasmodium* species but were identified as *P. knowlesi* infections by PCR, were also included. Case records were reviewed, and clinical information was entered into a standardized data collection form. Severe disease was classified on the basis of WHO criteria for severe falciparum malaria (*22*). National policy recommends that all patients with microscopy-diagnosed malaria be hospitalized until negative blood smears are obtained on 2 consecutive examinations. The study was approved by the Medical Research Ethics Sub-Committee of the Malaysian Ministry of Health and the Menzies School of Health Research, Australia.

### Laboratory Procedures

Blood films were examined by experienced laboratory microscopists, and the parasite count was classified on a scale of 1 to 4 (1= 4–40 parasites/µL, 2 = 41–400 parasites/µL, 3 = 401–4,000 parasites/µL, 4 = >4,000 parasites/µL). Hematologic results (Sysmex XT1800 [Sysmex Corp., Mundelein, IL, USA] and CELL-DYN Sapphire [Abbott Diagnostics, Abbot Park, IL, USA]) and prothrombin and partial thromboplastin times (STA Compact Hemostasis Analyzer [Diagnostica Stago, Asnières sur Seine, France]) were obtained on site. Serum sodium, potassium, glucose, creatinine, bilirubin, albumin (Roche/Hitachi Modular Analytics EVO, Roche, Basel, Switzerland), and arterial blood gas levels (Radiometer ABL520, Radiometer, Brønchøj, Denmark) were also assayed on site. Blood cultures were performed with an automated system (Becton Dickinson, Franklin Lakes, NJ, USA) and dengue serology by ELISA (PanBio, Brisbane, Australia). In accordance with QEH policy, all slides indicating *P. malariae* monoinfection or mixed infections were sent for molecular testing at the Sabah State Reference Laboratory, along with ≈15% of other species. Parasite DNA was extracted, and nested PCR was performed for *P. falciparum*, *P. vivax*, *P. malariae*, *P. ovale*, and *P. knowlesi* by methods previously published (*1**,**17*).

### Statistical Analysis

Data were analyzed by using STATA version 9.2 (StataCorp LP, College Station, TX, USA). For continuous variables, intergroup differences were compared by Student *t* test or Mann-Whitney U test. For categorical outcome variables, intergroup differences were compared by using the χ^2^ test or Fisher exact test. Logistic regression was used to determine the association between binary outcomes and other variables. A 2-sided value of p<0.05 was considered significant.

## Results

### Baseline Characteristics

Included in the final analysis were 56 patients with PCR-confirmed *P. knowlesi* malaria. On the basis of WHO severity criteria (*22*), 22 (39%) had severe malaria, and 34 (61%) had uncomplicated disease (Figure). These patients were identified from a group of 74 patients with documented *P. malariae* malaria listed in the laboratory microscopy register: 54 had *P. knowlesi* monoinfection shown by PCR and medical records available for review (Figure). In addition, another 2 patients received a diagnosis by microscopy as being infected with other *Plasmodium* sp. but were found to have *P. knowlesi* monoinfections on PCR testing (Figure). All 24 patients with severe malaria had PCR performed; of these, 22 had *P. knowlesi* monoinfection (Figure). In the group with uncomplicated infection, 41 had PCR; 34/41 had only *P. knowlesi* monofection; 4 cases were mixed with *P. knowlesi* and other species; 2 cases were non–*P. knowlesi*, and no *Plasmodium* sp. was detected in 1 patient (Figure).

**Figure:**
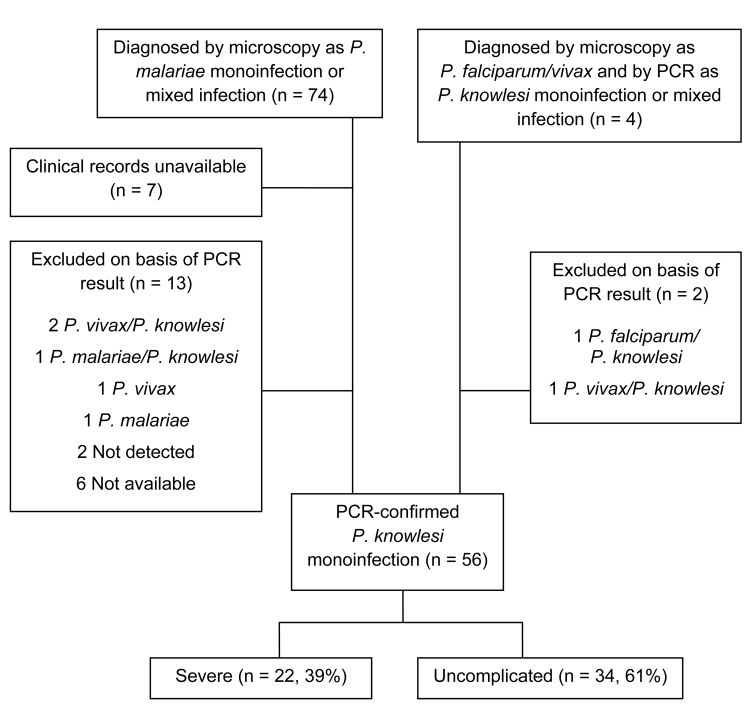
Flowchart of microscopy and PCR results of *Plasmodium malariae or P. knowlesi* infections by severity, Sabah, Malaysia, 2007–2009.

The baseline demographics and clinical symptoms of both groups with PCR-confirmed *P. knowlesi* monoinfections are detailed in Table 1. The mean age of the patients with severe malaria (57 years; 95% confidence interval [CI] 50–64 years) was significantly older than that of the uncomplicated group (37 years; 95% CI 34–42 years) (p<0.001); however, the proportion with chronic coexisting conditions did not differ significantly between groups (Table 1). In severe disease, the proportion of female patients (36%) was significantly higher than that in uncomplicated malaria (8.8%) (odds ratio [OR] 5.9, 95% CI 1.4–25.6; p = 0.02). Overall, 8 of 11 female patients had severe disease. Two women were pregnant; 1 had severe malaria, and the other had uncomplicated malaria. Two male patients had second uncomplicated infections during the study period; 1 became infected 10 months after the first, and the other had a mixed *P. vivax/P. knowlesi* infection 6 weeks after initial infection. Both patients had received chloroquine for the first *P. knowlesi* infection. One patient with uncomplicated disease also had HIV infection.

**Table 1:** Baseline demographic characteristics and clinical features of patients with severe and uncomplicated *Plasmodium knowlesi* malaria, Sabah, Malaysia, 2007–2009

Characteristic	Uncomplicated disease, n = 34	Severe disease, n = 22
Mean age, y* (range)	37 (20–66)	57 (22–84)
No. (%) female patients†	3 (8.8)	8 (36.3)
Self-reported previous malaria, no. patients	1	0
History of chronic illness, %	18	10
Mean duration of illness, d (range)	5.0 (1–30)	5.2 (2–7)
Symptoms		
Fever, %	100	96
Headache, %	62	57
Diarrhea, %	21	24
Physical findings		
Mean temperature (range)	38.2°C (36.5–41°C)	37.6°C (36.7–39.2 °C)
Mean respiratory rate, breaths/min* (range)	20 (14–26)	26 (15–50)
Mean pulse rate, beats/min (range)	95 (69–151)	100 (76–130)
Mean arterial pressure, mm Hg (range)*	85 (61–106)	74 (42–106)
Mean oxygen saturation, % (range)*	98 (92–100), n = 20	88 (56–100), n = 17

*p<0.05 by Mann Whitney U test. †p<0.05 by χ^2^ test.

Almost all patients with severe and uncomplicated disease had a history of fever, and no significant difference was found in duration of fever or other clinical symptoms before the patient sought treatment (Table 1). However, patients with severe complications had a lower mean arterial pressure and an increased respiratory rate. Of those not already intubated on transfer or admission to QEH, patients with severe disease also had lower oxygen saturation at room air than did those with uncomplicated disease (Table 1).

### Laboratory and Radiologic Investigations

In patients with severe disease, hemoglobin concentrations and platelet counts were significantly lower, and leukocyte counts, prothrombin time, and partial thromboplastin time were elevated, compared with results for patients with uncomplicated malaria **(**Table 2). Sodium and albumin concentrations were also significantly decreased, and creatinine and total bilirubin levels increased in patients with severe disease (Table 2). Parasite counts were significantly higher in severe disease (Table 2). Blood cultures, performed in 12/34 patients with uncomplicated disease, were all negative. Dengue serologic testing was performed on admission for 11 patients with uncomplicated disease; 10 were negative for immunoglobulin (Ig) M and IgG, and 1 patient was positive for IgM. Blood cultures were performed for 17 patients with severe disease; 1 patient had *Enterobacter cloacae* bacteremia, 5 had coagulase-negative *Staphylococcus* infection (attributed to contamination), and samples from the remainder were negative. Dengue serologic tests for 10 patients with severe disease were all negative. Chest radiographs were obtained on admission for 30 patients (8 with uncomplicated disease, 17 with severe disease); none with uncomplicated disease were reported to have infiltrates, compared with 8/17 with severe disease (p = 0.01); all of those with severe disease had acute respiratory distress syndrome (ARDS) with hypoxemia.

**Table 2:** Laboratory results for patients with severe and uncomplicated *Plasmodium knowlesi* malaria, Sabah, Malaysia, 2007–2009*

Laboratory result	Uncomplicated disease, n = 34	Severe disease, n = 22
Parasite count†	3 (1–4)	4 (2–4)
Hemoglobin concentration, gm/dL†	13.4 (9.1–17.6)	11.3 (6.2–16.8)
Leukocyte count, × 10^3^ cells/µL†	6.3 (3.4–15.3)	12.7 (3.5–21.6)
Neutrophil count, × 10^3^ cells/µL†	3.9 (1.4–10.1)	9.0 (2.73–16.9)
Lymphocyte count × 10^3^ cells/µL†	1.6 (0.6–3.3)	2.3 (0.24–6.4)
Prothrombin time, s†	13.8 (10.6–17.5)‡	14.9 (11.7–21)§
Partial thromboplastin time, s†	40 (29.9–57.6)‡	57 (31–136)§
Platelet count × 10^3^ cells/µL†	72 (21–227)	40 (12–130)
Serum creatinine, µmol/L†	92.6 (57–168)	289 (53–819)
Serum sodium concentration, mmol/L†	136 (128–146)	131 (123–140)
Serum total bilirubin, µmol/L	25.2 (4–48)¶	173 (11–660)
Serum albumin, gm/dL†	38 (26–73)#	22 (18–27)

*All values are mean (range). †p<0.05 by Student *t* test or Mann Whitney U test. ‡n = 23. §n = 19. ¶n = 15. #n = 13.

### Severe Malaria

Of patients with severe disease, 17 were referred from district hospitals and 5 were directly admitted. Twenty-one of these patients had complications on admission, and in 1 patient with acute renal failure, respiratory distress developed 3 days after the start of therapy. During the hospital course, 7 patients each had 1 WHO criterion for severity, and 15 each had >2 severity criteria (2, n = 6; 3, n = 5; 4, n = 1; 5, n = 1; and 6, n = 2) (Table 3). The mean age of the 6 patients who died was 64 years (95% CI 49–78 years), and the mean age of the 16 survivors (was 53 years (95% CI 45–61 years; p = 0.1). Decreased Glasgow Coma Scores (GCS 14 and 11) on initial hospital visit were seen in 2 patients who died, but other signs and symptoms did not satisfy WHO criteria for cerebral malaria. No cases of severe anemia (<6 g/dL) were found. Acute respiratory distress (n = 13), acute renal failure (n = 12), shock (n = 12), and hyperbilirubinemia (n = 9) were the most common manifestation of severe disease (Table 3). Seven patients had acidosis (on the basis of arterial blood gas analysis) (Table 3). Seven (32%) patients had significant elevations of the prothrombin and partial thromboplastin times, although none were reported to have clinically important bleeding. In patients with acute respiratory distress, the ratio of the partial pressure of oxygen to the fraction of inspired oxygen (PaO_2_:FiO_2_) was available for 11/13 patients. In this group, the mean ratio was 165 (range 101–250), with 10 meeting the criteria for ARDS (PaO_2_:FiO_2_ <200). Eight patients had cardiac function evaluated by transthoracic echocardiogram, and all had normal results, except for a 76-year-old woman with a left ventricular ejection fraction of 30%.

**Table 3:** Details of patients with severe *Plasmodium knowlesi* malaria, Sabah, Malaysia, 2007–2009*

Patient no.	Age, y/ sex	Severity†	Parasite count	Platelets/ µL	Blood products	ICU	Inotropes	Ventilation	Dialysis	Treatment	Outcome
*P. knowlesi* only											
1	76/M	1, 6, 7	3	17,000	Plt	Y	N	N	N	Artesunate	S
2	22/M	4, 7	3	38,000	Bld	Y	Y	Y	N	Quinine	S
3	29/M	2	4	16,000	Bld, Plt	Y	N	Y	Y	Quinine	S
4	76/F	1, 2, 3, 4, 6, 7	4	42,000	Bld	Y	Y	Y	Y	Quinine	D
5	50/M	2, 4	2	60,000	Bld	Y	Y	N	Y	Quinine	S
6	55/M	2, 4, 7	4	42,000	N	Y	Y	N	N	Quinine	S
7	49/M	1	4	78,000	N	Y	N	N	N	Quinine	S
8	38/F	2	4	29,000	Bld	Y	N	N	Y	Quinine	S
9	65/M	3, 4	3	17,000	N	N	N	N	N	Quinine	S
10	70/F	2, 4, 6, 7	4	48,000	Bld	Y	Y	Y	Y	Quinine	S
11	65/F	1, 2, 4, 6, 7	4	26,000	Bld, FFP	Y	Y	Y	Y	Quinine	D
12	75/F	4, 6	3	22,000	N	N	Y	N	N	Artesunate	S
13	69/F	7	2	130,000	N	N	N	N	N	Artesunate	S
14	57/M	1, 2, 3, 4, 6, 7	4	35,000	Bld, Plt, FFP	Y	Y	Y	Y	Artesunate	D
15	60/M	1	4	37,000	N	N	N	N	N	Quinine	S
16	44/M	1	3	61,000	N	N	N	N	N	Artesunate	S
17	54/M	4, 7	2	72,000	N	Y	Y	N	N	Quinine	S
18	69/M	1, 2	3	41,000	Bld	Y	N	N	Y	Quinine	S
19	84/F	2, 5, 7	2	34,000	N	N	N	Y	N	Quinine	D
20	54/F	2, 4, 7	3	12,000	Bld, Plt	Y	Y	Y	Y	Artesunate	S
21	56/M	4, 7	4	28,000	N	N	Y	Y	N	Quinine	D
22	46/M	1, 2, 7	4	16,000	N	Y	Y	Y	Y	Quinine	D
*P. vivax* + *P. knowlesi*	48/M	1, 2, 6	2	24,000	N	Y	N	N	Y	Artesunate	S
ND	28/M	7	3	80,000	N	Y	N	Y	N	Artesunate	S

*ICU, intensive care unit; Bld, blood; Plt, platelets; Y, yes; N, no; S, survived; D, died; FFP, fresh frozen plasma; ND, none detected. †Severity criteria: 1, hyperbilirubinemia; 2, acute renal failure; 3, hypoglycemia; 4, shock; 5, blackwater fever; 6, acidosis; 7, respiratory distress.

Seventeen patients required intensive care unit management, 12 received inotrope support, 11 required hemodialysis, and 10 received mechanical ventilation (Table 3). The median duration of intensive care stay was 6 days (range 1–11 days); for hemodialysis, 3 days (range 1–6); and for mechanical ventilation, 3 days (range 1–9). Eleven patients were transfused with erythrocytes, 2 with fresh frozen plasma, and 4 with platelets (Table 3).

### Malarial and Antimicrobial Drug Therapy

Of 34 patients with confirmed, uncomplicated *P. knowlesi* malaria, 15 received oral chloroquine, 11 received oral quinine, and 8 received artemether-lumefantrine. Two patients from the quinine group received intravenous quinine for ≈24 hours before treatment was changed to oral therapy. Daily peripheral blood films were available for 10 patients who received chloroquine (mean admission parasitemia 2+), for 8 who received quinine (mean admission parasitemia 3+), and for 6 who received artemether-lumefantrine (mean admission parasitemia 2+). When we excluded patients who received intravenous therapy initially, the difference was significant in median parasite clearance times between those who received artemether-lumefantrine (1 day; range 0–3) and those who received chloroquine (2.5 days; range 1–3) or quinine (2.5 days; range 1–3); p = 0.01. The proportion with negative results for parasitemia by day 1 was 4/6, 3/10, and 1/8 for artemether-lumefantrine, chloroquine, and quinine, respectively (p = 0.1), and 5/6, 5/10, and 4/8 for each group on day 2 (p = 0.2). Among patients with uncomplicated disease, 11/34 patients received doxycycline, and 7/34 received other antimicrobial drugs during their hospitalization.

In December 2008, hospital policy changed, and the recommendation was made that patients receive intravenous artesunate, when available, rather than quinine for treatment of severe malaria. Of the 22 patients with severe *P. knowlesi* malaria, 16 received intravenous quinine, and 6 received intravenous artesunate. Daily peripheral blood films were available for 11 of the patients in the quinine group (mean admission parasitemia 3+) and 4 of the artesunate group patients (mean admission parasitemia 3+), with median parasite clearance time significantly shorter with artesunate (2 days; range 1–3) than with quinine (4 days; range 2–7) (p = 0.02). Of the 6 patients who died, 5 received quinine (median severity criteria 2; case-fatality rate 31%), and 1 received artesunate (median severity criteria 2.5; case-fatality rate 16.6%). Of patients with severe malaria, 13/22 received doxycycline, and 16/22 received other antimicrobial drugs during their hospital course.

### Outcome

Six (27%) of the 22 patients with severe malaria died; mean time from admission to QEH and death was 2.5 days (range 0–4). Of these, all had >3 severity criteria; 6 had ARDS, 5 had acute renal failure, and 4 had shock. All patients who died had a parasitemia level of 4+; survivors had a median level of 3+. None of the patients with uncomplicated disease died. The mean duration of hospital stay was 8.4 days (95% CI 6.3–10.5) for those with severe disease and 5.3 days (95% CI 4.1–7.4) for those with uncomplicated malaria.

## Discussion

Studies from Sarawak have shown that *P. knowlesi* infections can result in severe and fatal disease (*2**,**17*). The present 2-year case series from Sabah indicates that in a tertiary referral hospital setting, the proportion of severe *P. knowlesi* malaria is higher than reported previously, with 39% of patients having severe malaria according to WHO criteria. The increased frequency of severe disease likely reflects referral bias because a large proportion of patients were referred from surrounding district hospitals. The case-fatality rate for severe malaria of 27% in this study is comparable to that of a previous study (*2*) and at least as high as that seen with *P. falciparum* malaria (*21*). The main demographic factors for severe malaria were increasing age (mean 57 years) and female gender. The former is consistent with a study of falciparum malaria where age was an independent risk factor for development of severe disease and death (*23*). The reason(s) for the large proportion of severe disease in female patients, noted previously in knowlesi malaria (*2*) and vivax malaria (*24*), remain unclear.

For 12 of the 13 patients with respiratory distress, the diagnosis was confirmed by a low arterial partial pressure of oxygen and decreased oxygen saturation with a need for mechanical ventilation. One patient with an increased respiratory rate alone had metabolic acidosis; the 4 other patients had ARDS and hypoxemia. This finding suggests that hypoxemia from acute lung injury is the major cause of respiratory distress in *P. knowlesi* malaria, although metabolic acidosis can also contribute. Shock occurred in more than half of patients who had severe malaria; however, repeated blood cultures showed clinically significant bacteremia in <10% of patients, which suggests that in most cases of severe knowlesi malaria, concurrent bacteremia does not contribute to hypotension. A previous report with smaller numbers of severe knowlesi malaria found metabolic acidosis in only 10% (*2*), compared with 30% of severe patients in this study for whom arterial blood gas results showed clear metabolic acidosis. The cause in 6 of 7 patients with acidosis may have been shock and hypoxemia; only 1 patient had neither. Although 30% of patients with severe disease had elevated prothrombin time or partial thromboplastin time, no clinically notable bleeding episodes were observed.

The susceptibility of pregnant women to severe disease in falciparum malaria (*22*) may also be the case in knowlesi malaria; 18% of women admitted with *P. knowlesi* malaria were pregnant. One patient in the third trimester of pregnancy survived acute renal failure and shock, but the fetus died.

The multiorgan failure experienced by patients with severe knowlesi malaria is similar to that reported in adults with severe falciparum malaria in areas where the transmission rate is low and unstable (*22*). However, as seen in a smaller series of severe knowlesi malaria, the ≈50% proportion with ARDS and shock is higher than that reported in series of severe falciparum malaria (*22**,**23**,**25**–**29*). Furthermore, 2 of the major clinical syndromes of severe falciparum malaria—unarousable coma and severe anemia—were absent (*2*). The absence of severe anemia may reflect the lower malaria transmission rate, the relatively short duration of illness, and the exclusion from this adult referral hospital of children, an age group prone to this complication in falciparum malaria (*23*). Severe anemia has been described in knowlesi malaria in children elsewhere in Sabah (*30*). The reasons for the lack of coma are less clear and may reflect differences in pathophysiology between knowlesi and falciparum malaria. In the only detailed autopsy study of fatal knowlesi malaria, widespread microvascular parasite accumulation was found, including within the brain, but no features to suggest cytoadherence of parasitized red cells to endothelial cells, a hallmark of the pathophysiology of severe falciparum malaria (*31*). Additional causes of impaired microvascular flow and organ dysfunction in falciparum malaria include dysregulated immune responses (*32*), endothelial activation with elevated angiopoietin-2 and von Willebrand factor (*33**,**34*), and decreased vascular nitric oxide bioavailability (*25*) and red cell deformability (*35*), but their roles in knowlesi malaria remain unknown.

Thrombocytopenia is nearly universal in *P. knowlesi* infections; platelet counts are lowest in cases of severe disease when no evidence suggests concurrent dengue. In contrast to platelet counts, leukocyte counts were higher in severe malaria than in uncomplicated malaria. Secondary bacterial infection was uncommon, which suggests that severe *P. knowlesi* infection itself may account for the neutrophilia.

The optimal management of knowlesi malaria is not known, and the 2010 WHO Malaria Treatment Guidelines do not provide recommendations for its treatment (*19*). Artemisinin-derivative combination therapy is recommended as first-line treatment of falciparum malaria in Africa and Asia, but there are no reports of artemisinin-derivative combination therapy efficacy in knowlesi malaria. In previous reports, uncomplicated *P. knowlesi* malaria was treated with chloroquine and primaquine (*2**,**36*), whereas severe disease was treated with intravenous quinine (*1**,**2**,**17*). Past studies in Sarawak have shown that although most patients with microscopy-diagnosed *P. malariae* infection had *P. knowlesi*, ≈10% were actually infected with *P. falciparum* (*17*). In settings such as Malaysia, with a high prevalence of chloroquine-resistant falciparum malaria (*37**,**38*), inadvertent use of chloroquine for misdiagnosed falciparum malaria may have deleterious consequences. In uncomplicated knowlesi malaria, we found that chloroquine, quinine, and artemether-lumefantrine were all efficacious, and although comparisons were uncontrolled, those receiving artemether-lumefantrine had faster parasite clearance times. The 1-day median parasite clearance time after using artemether-lumefantrine in our hospitalized patients was similar to that seen in a community study in which chloroquine was used (*2*), though parasite densities may not have been comparable.

Intravenous artesunate (compared with quinine) reduces the proportion of deaths in severe falciparum malaria (*21*), but its efficacy is unknown in severe knowlesi malaria. In our study, artesunate-treated patients had faster parasite clearance, and the case-fatality rate (17%) was lower than for those who received quinine (31%). However, the retrospective design and small number of severe cases and deaths do not enable us to assess a possible survival benefit. Current treatment policy at QEH for uncomplicated knowlesi malaria is oral artemether-lumefantrine and for severe knowlesi malaria, intravenous artesunate.

Our study has several limitations. The main ones are the retrospective design and inability to review 10% of the charts. Although samples from most *P. malariae* patients were sent for molecular confirmation, 8% of the results were unavailable. Several cases microscopically diagnosed as falciparum or vivax malaria were PCR-positive for *P. knowlesi*. Because only ≈15% of non–*P. malariae* slides are sent for PCR, we may have underestimated the true proportion of patients hospitalized with knowlesi malaria. Because QEH is a hospital for adults, we were unable to describe the disease spectrum in children.

Our study further highlights the public health implications of *P. knowlesi*. A high proportion of knowlesi malaria patients admitted to a tertiary care referral hospital in a malaria-endemic area had severe and fatal disease characterized by multiorgan failure, a high proportion with ARDS and shock, and a notable absence of coma. The pathogenic mechanisms underlying this disease spectrum remain unknown. Artemisinin derivatives result in rapid parasite clearance and are efficacious in both uncomplicated and severe knowlesi malaria. Prospective studies to further define the epidemiology, pathogenesis, and optimal treatment for knowlesi malaria are needed.
